# First Molecular Verification of the Two-Spot Cotton Leafhopper *Amrasca biguttula* (Hemiptera: Cicadellidae) in the United States

**DOI:** 10.3390/insects17030313

**Published:** 2026-03-13

**Authors:** Chaoyang Zhao, Kipling S. Balkcom

**Affiliations:** USDA-ARS, National Soil Dynamics Laboratory, Auburn, AL 36832, USA

**Keywords:** cotton jassid, invasive species, mitochondrial DNA, molecular identification, cotton agroecosystem

## Abstract

This report contains the first molecular record of the two-spot cotton leafhopper, *Amrasca biguttula* (Ishida) (Hemiptera: Cicadellidae), in the United States. Nymphs of multiple instars and adult specimens were collected from a cotton (*Gossypium hirsutum*) field in Macon County, Alabama, in August 2025. While distinct paired dark spots were observed on the forewings of adult specimens, this trait was inconsistently present on nymphal wing pads. *Cytochrome oxidase I* (COI) DNA barcoding confirmed the specimen identity. The United States sequence shared > 99% identity with Asian *A. biguttula* references, and phylogenetic analysis placed it within the *A. biguttula* clade with 100% posterior probability support. Although this pest was previously reported in 2023 from Puerto Rico based solely on morphological traits, our findings provide the first DNA-confirmed evidence of its presence in the United States. Given its well-documented role in damaging cotton across Asia and Africa, this report underscores the urgent need for monitoring and development of management strategies in United States cotton-growing regions.

## Simple Summary

The two-spot cotton leafhopper (*Amrasca biguttula*) is a destructive insect pest that causes severe yield losses in cotton and other crops across Asia and Africa. Native to Asia, this species was recently reported in the Western Hemisphere based on morphological features, but its presence in the United States had not been confirmed using DNA methods. In this study, we observed *A. biguttula* activity in a cotton field in Macon County, Alabama, USA, collected specimens across multiple life stages, and confirmed its identity using DNA barcoding. These results represent the first molecular confirmation of *A. biguttula* in the United States. Accurate identification is critical because this species closely resembles other leafhoppers, especially in immature stages. Our findings provide a reliable genetic reference to support monitoring and management efforts in U.S. cotton production systems.

## 1. Introduction

The two-spot cotton leafhopper, *Amrasca biguttula* (Ishida), also known as the cotton jassid or Indian cotton leafhopper, is a significant insect pest that feeds on a wide range of hosts, with a primary preference for malvaceous crops such as cotton (*Gossypium* spp.) and okra (*Abelmoschus esculentus*) [1]. Both nymphs and adults feed on the undersides of leaves, extracting plant sap and introducing salivary toxins. This feeding behavior induces “hopperburn”, characterized by yellowing, necrotic spotting, leaf curling, and eventual defoliation. Severe infestations can result in yield losses exceeding 60% in cotton and 50% in okra [2,3].

Native to Asia, this species was first reported in the Western Hemisphere in 2023, based on morphological identification of specimens collected in Puerto Rico [4]. In 2024 and 2025, extension bulletins and pest alerts reported the presence of *A. biguttula* in Florida, Georgia, South Carolina, and Alabama, warning growers and researchers of the potential threat, and its occurrence in the southeastern United States was recently summarized [5]. However, these reports were based solely on morphological identification, and the presence of *A. biguttula* in the United States had not previously been confirmed using molecular techniques.

Because *A. biguttula* closely resembles other Empoascini leafhoppers, particularly in immature stages, molecular diagnostics are essential for accurate species verification. In this study, we collected specimens from a cotton field with suspected *A. biguttula* infestation and confirmed their identity using DNA-based methods. This work provides a validated molecular reference to support future diagnostic and surveillance efforts.

## 2. Materials and Methods

### 2.1. Insect Collection

In August 2025, “hopperburn” symptoms were observed in a cotton (*G. hirsutum*) field at the E.V. Smith Research Center, Auburn University, Macon County, Alabama (32.420922° N, 85.887618° W). More than 10 symptomatic plants located along the edge of the field were examined. Leaves from the upper one-third of plants containing nymphs and adults of different life stages were collected from several plants and placed in plastic zip-lock bags for transport to the laboratory. In the laboratory, insects were removed from the leaves and preserved in 100% ethanol for photographic documentation and subsequent molecular analysis.

### 2.2. DNA Extraction and COI Amplification

Genomic DNA was extracted from two nymphs and one adult using the DNeasy Blood & Tissue Kit (Qiagen, Hilden, Germany). A 658-bp barcoding fragment of the mitochondrial *cytochrome oxidase subunit I* (COI) gene was amplified using Platinum^TM^ Taq DNA polymerase (Thermo Fisher Scientific, Waltham, MA, USA) with primers LCO1490 and HCO2198 [6]. PCR conditions were as follows: initial denature at 94 °C for 5 min; 35 cycles of 94 °C for 30 s, 49 °C for 30 s, and 72 °C for 1 min; and a final extension at 72 °C for 8 min. Amplicons were verified on agarose gel for specificity, ligated into the pCR4-TOPO^®^ TA vector, and transformed into competent TOP10 cells (Thermo Fisher Scientific, Waltham, MA, USA). Colony PCR was performed to identify positive clones, plasmid DNA was isolated, and insert sequences were determined by Sanger sequencing.

### 2.3. Sequence Analysis and Phylogeny

Sequences were compared against the GenBank nucleotide database using BLASTN (https://blast.ncbi.nlm.nih.gov/Blast.cgi, accessed on 30 August 2025). For phylogenetic analysis, representative sequences of *A. biguttula* were aligned using MAFFT (v7.505) with the ‘auto’ setting [7], and the alignments were trimmed with TrimAl (v1.2) based on a gap threshold of 0.25 [8]. The best-fit substitution model (GTR+G) was selected based on Bayesian Information Criterion implemented in MEGA11 [9]. Phylogenetic inference was performed in MrBayes (v3.2.7) [10] with two runs of four chains each, continuing until the standard deviation of split frequencies fell below 0.01. The first 25% of generations were discarded as burn-in, and the remaining samples were used to construct a 50% majority-rule consensus tree.

## 3. Results and Discussion

**Figure 1:**
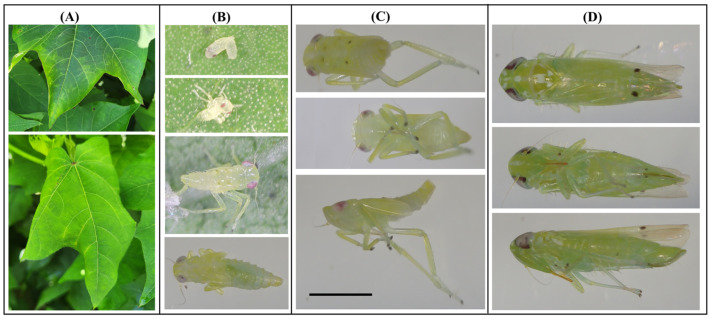
Morphology of *Amrasca biguttula* and associated cotton leaf damage. (**A**) Cotton leaves showing “hopperburn” symptoms: discoloration (**top**) and curling (**bottom**). (**B**) Nymphal stages: newly hatched (**top**), first instar (**second from top**), second or later instar (**second from bottom**), and third or later instar without wing-pad spots (**bottom**). (**C**) Later nymphal instar with wing-pad spots in dorsal (**top**), ventral (**middle**), and lateral (**bottom**) views. (**D**) Adult with forewing spots in dorsal (**top**), ventral (**middle**), and lateral (**bottom**) views. All insect images (**B**–**D**) are scaled to the reference bar (1 mm) in panel (**C**).

Cotton leaves exhibited characteristic “hopperburn” symptoms, including discoloration and curling (Figure 1A). Nymphs of multiple instars and adults were observed on affected plants (Figure 1B–D). Adults displayed paired dark spots at the tips of their forewings (Figure 1D), whereas pigmentation on nymphal wing pads was inconsistently expressed (Figure 1B,C). Notably, the pair of small black spots typically found preapically on the crown of the head were absent in our specimens. Pigmentation characters such as these spots may vary among individuals or may sometimes be faint or absent, as noted in diagnostic resources for *A. biguttula* [11]. Nymph body length varied by instar, while adults measured approximately 3 mm in length. Although these features supported a preliminary identification, the morphological similarity of *A. biguttula* to related Cicadellidae species and the absence of reliable diagnostic characters in certain life stages, particularly immature stages and female adults, required molecular confirmation, as morphological identification relies primarily on forewing spots and male genitalia that are only present or diagnostic in adult males [4].

All three individuals—two nymphs (one with wing-pad pigmentation (Figure 1C) and one without (Figure 1B, bottom) and one adult—yielded identical 658-bp COI sequences (GenBank accession PX247763). A BLAST search of this sequence against the GenBank core nucleotide database returned *A. biguttula* COI sequences as the top hits. Sequence alignment showed >99% identity between the U.S. sequence and samples collected in India, China, and Pakistan (Figure S1). Because this study focuses on species verification rather than population-level analysis, the COI sequences obtained from the examined specimens were sufficient to confirm species identification. The identical sequences obtained from three specimens represent a single haplotype in this collection.

**Figure 2:**
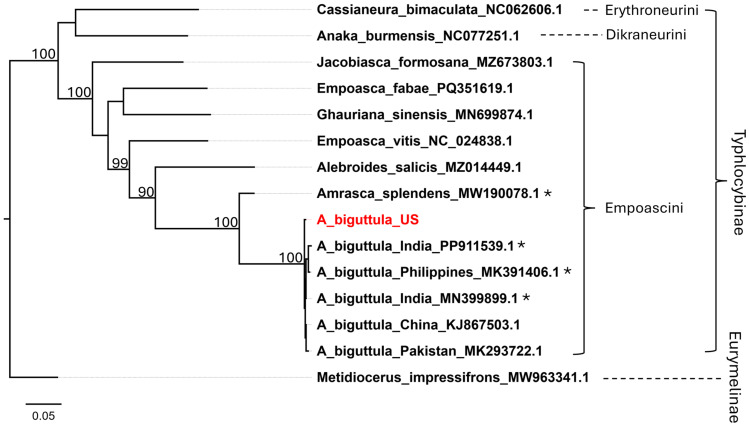
Phylogenetic placement of the U.S. *Amrasca biguttula* isolate within Asian lineages. Bayesian phylogeny of the COI barcoding region of *A. biguttula*, including the United States isolate (highlighted in red) and representative Asian sequences, alongside other species from the subfamily Typhlocybinae. The tree is rooted with *Metidiocerus impressifrons* (subfamily Eurymelinae). Taxon labels include species names followed by their corresponding GenBank accession numbers. Sequences labeled with an asterisk (*) indicate specimens that were morphologically identified. Posterior probabilities > 60% are shown at nodes. Scale bar represents substitutions per site.

To resolve the phylogenetic relationship between the Alabama specimens and Asian *A. biguttula* sequences, including those from morphologically identified specimens such as MK391460 (Philippines) [12], MN399899.1.1 (Vietnam) [13], and PP11539.1.1 (India) [14], a Bayesian phylogenetic analysis was conducted using their COI sequences. The resulting topology placed the Alabama sequence within the *A. biguttula* clade with 100% posterior probability support (Figure 2). This placement distinguished it from other *Amrasca* species (e.g., *A. splendens* morphologically identified by [13]) and from non-*Amrasca* taxa within the tribe Empoascini.

Despite its confirmed presence in the cotton field where *A. biguttula* was collected, we did not observe obvious yield losses at the time of sampling during the 2025 season. This likely reflects the early stage of invasion in this location: the pest was detected after cotton had fruited, and the population had not yet propagated to high densities. Following collection, an insecticide was sprayed to mitigate potential yield damage. While current yield impacts appeared minimal, continued surveillance will be necessary to assess its potential to cause economic losses. Populations may increase in future seasons, which will require confirmation through accurate species identification.

Identifying *A. biguttula* adults is relatively easy due to the presence of characteristic forewing spots. However, this feature is inconsistently expressed in nymphs, which are more commonly collected during field scouting because of their limited mobility. Furthermore, cotton and other host plants may be co-infested by *A. biguttula* together with morphologically similar Empoascini species, such as the potato leafhopper *Empoasca fabae* [15], making morphological diagnosis of nymphs unreliable. In such cases, molecular diagnostics offer a dependable approach that complements traditional morphological identification, allowing reliable species discrimination and improved detection of mixed infestations to support appropriate mitigation strategies [16].

Molecular confirmation of *A. biguttula* in this study, based on samples collected in Alabama, provides the first molecularly validated record for the United States and serves as a valuable reference for regulatory agencies, researchers, and extension specialists. Although extension reports indicate that the pest is present in several southern states, the extent of its establishment and impact in United States cotton systems remains uncertain. Given its broad host range [1], coordinated surveillance, along with studies on management tactics such as cultural practices, biological control, and insecticide efficacy, will be essential for developing effective integrated pest management (IPM) strategies.

## Data Availability

The COI sequence from the Alabama *A. biguttula* specimens has been deposited in GenBank under accession number PX247763. A preprint version of this manuscript was first made publicly available on 7 September 2025, via bioRxiv https://www.biorxiv.org/content/10.1101/2025.09.03.673823v1, and was last accessed on 1 February 2026.

## Supplementary Materials

The following supporting information can be downloaded at https://www.mdpi.com/article/10.3390/insects17030313/s1, Figure S1: MAFFT alignment of the COI barcoding region of four Amrasca biguttula isolates.
